# From “unhappy” to “happy” with vortioxetine: how effective psychoeducation, illness awareness, and safe medications can provide patients with a degree of autonomy in managing their condition. A case report

**DOI:** 10.1192/j.eurpsy.2025.1065

**Published:** 2025-08-26

**Authors:** M. Menendez Munoz, H.-B. J. Min Kim, E. Lopez Bardon

**Affiliations:** 1 SERVICIO DE PSIQUIATRIA, COMPLEJO ASISTENCIAL UNIVERSITARIO DE LEON, LEON; 2 SERVICIO DE PSIQUIATRÍA, COMPLEJO ASISTENCIAL UNIVERSITARIO DE LEÓN, LEÓN, SPAIN

## Abstract

**Introduction:**

We present the case of a 61-year-old retired woman with hypothyroidism and rheumatoid arthritis who was diagnosed with bipolar disorder in 2006 after a manic episode. Her initial treatment included venlafaxine, valproic acid, quetiapine, zolpidem, and lormetazepam. She had several manic episodes over the years, some requiring hospitalization. In 2017, venlafaxine was replaced with vortioxetine, which she now introduces when detecting depressive phases, under psychiatric supervision but with some autonomy. She has remained stable, with occasional manic or hypomanic episodes triggered by stress, but none requiring hospitalization.

Figures 1 and 2 illustrate the patient’s self-perception changes with vortioxetine treatment. Figure 1 shows her as unhappy during the depressive phase (top) and happy after recovery (bottom). Figure 2 depicts her self-image during the depressive phase (left) and after recovery (right).

**Objectives:**

- To assess the effectiveness, safety and risk of mood swings of vortioxetine in a patient with bipolar disorder during depressive phases.
- To determine if effective psychoeducation allows patients to manage some of their medications safely under specialist supervision.

**Methods:**

The patient mantains treatment with vortioxetine 20 mg daily, valproic acid 500 mg every 12 hours, quetiapine extended release 400 mg at dinner, zolpidem 15 mg, and lormetazepam 2 mg at bedtime during depressive phases. In manic episodes, quetiapine 300 mg is added, vortioxetine is discontinued, and the dosage of hypnotics is doubled. Intensive psychological support has improved her disease awareness and treatment adherence, allowing her to adjust vortioxetine (but not other medications) as needed under medical supervision. She has not reported any adverse effects from vortioxetine.

**Results:**

Vortioxetine 20 mg could effectively treat depressive phases without causing mania. Though research on its use in bipolar disorder is limited, it shows potential when combined with a mood stabilizer, with a response time of about nine weeks and a low risk of hypomania/mania. It may also help with cognitive decline and brain inflammation related to bipolar disorder, improving cognitive performance and reducing inflammatory markers. Vortioxetine is noted for its effectiveness, tolerability, and low dropout rate.

**Image 1:**

**Figure:**
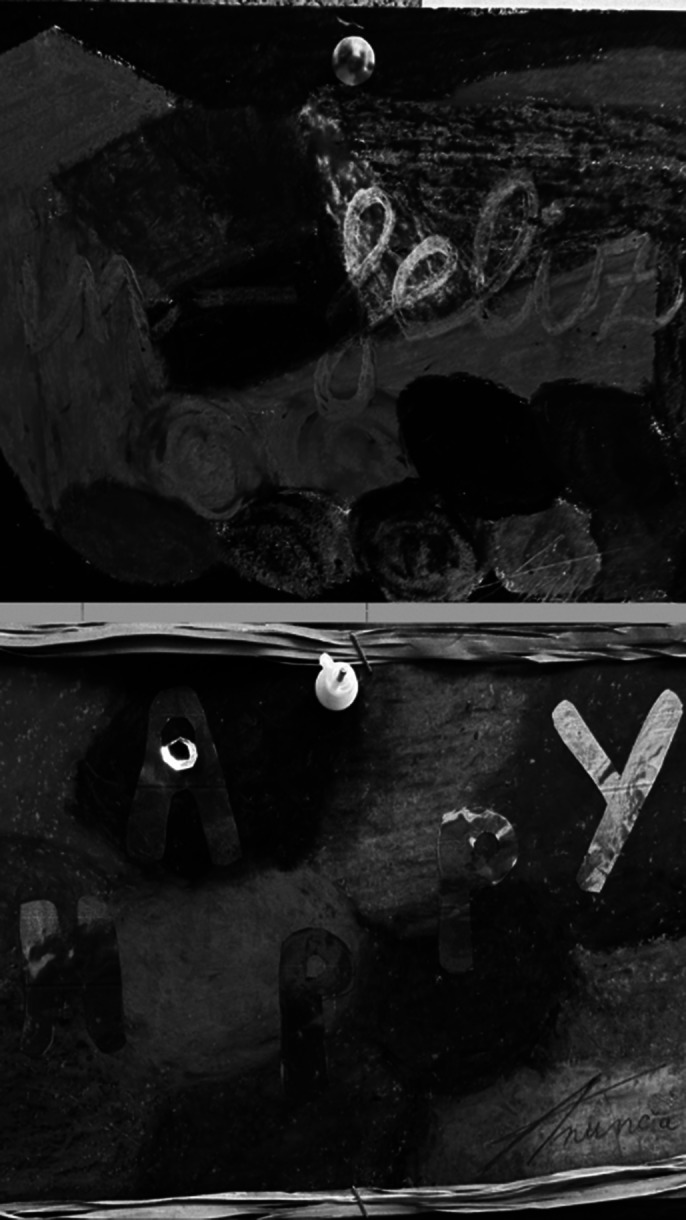

**Image 2:**

**Figure:**
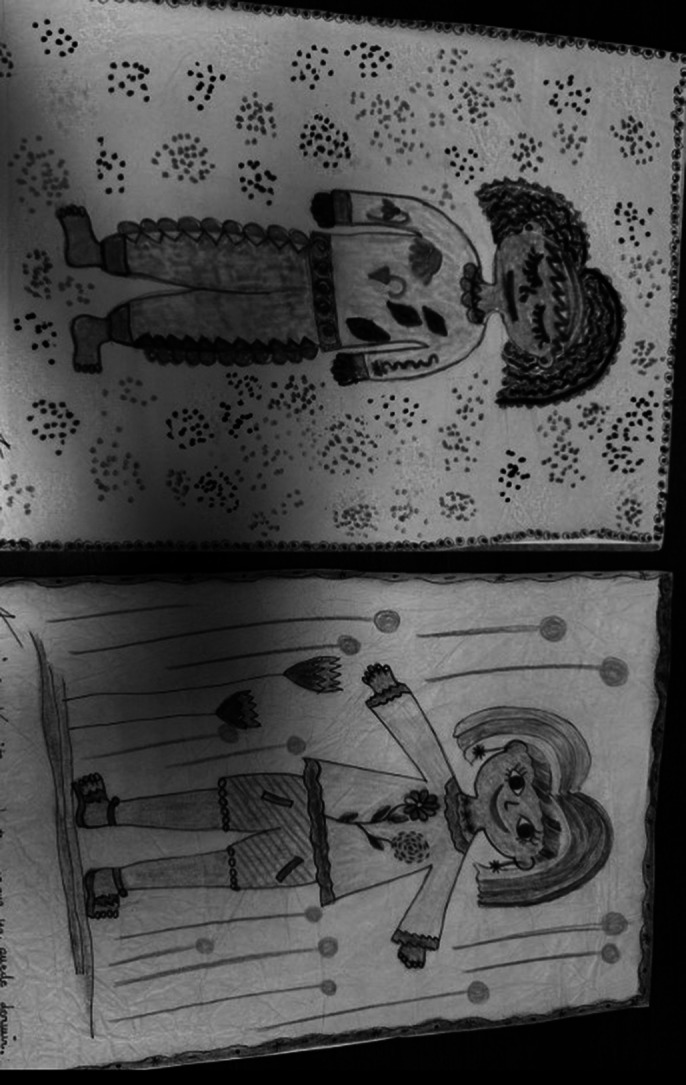

**Conclusions:**

Vortioxetine 20 mg may be effective for treating depressive phases in bipolar disorder with a lower risk of manic episodes compared to other antidepressants. Its procognitive and potentially anti-inflammatory effects could also support stability in non-psychiatric comorbidities. For this patient, good psychoeducation has facilitated a degree of independence in managing the medication, which is aided by vortioxetine’s safety and ease of use for both professionals and patients.

**Disclosure of Interest:**

None Declared

